# Industrial structure, high-quality development of logistics industry and the economy

**DOI:** 10.1371/journal.pone.0285229

**Published:** 2023-05-17

**Authors:** Borui Yan, Bo Yao, Chenjing Zhang

**Affiliations:** School of Economics and Management, Xianyang Normal University, Xianyang, China; Shenzhen University, CHINA

## Abstract

The logistics industry is closely related to the high-quality economic development. At different levels of industrial structure, the relationship between high-quality development of the logistics industry and the high-quality economic development will vary, resulting in different roles and paths in promoting economic development. However, there is still a lack of research on the relationship between high-quality development of the logistics industry and high-quality economic development at different levels of industrial structure, and further empirical research is needed. It used the benchmark regression model to analyze the impact of the high-quality development of the logistics industry on high-quality economic development, and the panel threshold model was used to analyze the impact of the logistics industry on high-quality economic development at different levels of industrial structure development. The results show that the high-quality development of the logistics industry has a positive role in promoting the high-quality economic development, and in different levels of industrial structure development, the impact of high-quality development level of logistics industry on the high-quality economic development is different. Therefore, it is necessary to further optimize the industrial structure, promote the deep integration and development of logistics and related industries, and continue to promote the high-quality development of the logistics industry. And when formulating development strategies for the logistics industry, governments and enterprises need to consider factors such as changes in industrial structure, the overall goals of national economy, people’s livelihood, and social development, in order to provide solid support for achieving high-quality economic development. This paper demonstrates the importance of high-quality development of the logistics industry in high-quality economic development, and it encourages the adoption of different strategies at different stages of industrial structure development to promote high-quality development of the logistics industry, and achieve high-quality economic development.

## Introduction

China is currently in a critical period of green economy transformation and upgrading, promoting high-quality economic development has become an opportunity and challenge for China [1]. High-quality economic development is a necessary way to ensure stable socio-economic growth and sustainable development. Meanwhile, China has become the largest logistics market in the world and has developed into a logistics powerhouse that has global influence. Logistics is a fundamental service industry for socio-economic development and a pillar industry of the national economy, the higher the level of logistics development, the greater the market activities and the faster the socio-economic development [1]. The logistics industry is widely recognized as an accelerator of economic development and the third source of profits [2]. It has gradually emerged as one of the pillar industries that support urban economic development [3]. Based on China’s energy structure, the logistics industry has become one of the largest and most important energy consumers and carbon emission contributors in China, and a key industry for environmental management and supervision. According to the fifth evaluation report of IPCC, without changing the development of high energy consumption in the logistics industry, its energy consumption will increase by 80% by 2030. Moreover, China’s economic and social development relies heavily on transportation, and a large amount of energy consumption produces a large amount of carbon emissions [4, 5]. The pressure on energy conservation and emission reduction in China’s logistics industry has increased significantly, and mitigating environmental problems in China is very important for global ecology [6]. The contradiction between the rapid development of the logistics industry and environmental pollution needs to be properly addressed. The logistics industry can play an important influence in reducing energy consumption and carbon emissions to achieve high-quality development [7].

Although many scholars have extensively researched the high-quality development of the economy and logistics industry, the nature of the logistics industry makes it impossible to achieve high-quality development in isolation. This is because logistics covers various areas, including micro-enterprises, mesoscopic industries, macro-regional and international logistics activities. Moreover, the scope of key industries that the logistics industry serves is a crucial factor that changes with different industrial structures. At different stages of high-quality economic development, the industrial structure will undergo corresponding changes. The differences in the various stages of such development are mainly reflected in the characteristics and composition of the industrial structure. In the initial stage, the economy is mainly based on agriculture and traditional crafts, and the industrial structure mainly relies on labor-intensive primitive resources. With the passage of time and the development of technology, the economy enters the medium to high stage and modernization stage, and correspondingly, the industrial structure will also undergo corresponding changes. The modern industrial structure focuses on the development of strategic and technologically advanced manufacturing, modern service, digital economy and other industries, which will involve more diverse and diverse industries and fields. The relationship between high-quality development of the logistics industry and high-quality economic development varies at different levels of industrial structure. In the case of a high proportion of the primary industry, logistics, as an important component of the circulation process, needs to pay high attention to the improvement of logistics facilities, logistics technology, and logistics service quality in order to effectively improve circulation efficiency and save circulation costs, and drive healthy economic development. When the proportion of the secondary industry is relatively high, the logistics industry, as an outward extension of the manufacturing industry, can play an important role in logistics distribution efficiency, supply chain management, logistics facility construction, and other aspects. When the industrial structure is improved and advanced manufacturing industries increase, the requirements for logistics services will also be higher, posing higher requirements and greater challenges for the high-quality development of the logistics industry. In the case of a relatively high proportion of the tertiary industry, the logistics industry, as an organic component of the service industry, is inseparable from other service industries such as retail, catering, agriculture, etc., forming the pattern of modern service industry. The quality of logistics development will directly affect the quality of services, and the service industry is the largest source of GDP growth, which will directly affect the high-quality development of the economy. The logistics industry also needs to continuously upgrade and innovate in different stages of high-quality economic development, meet the different needs and requirements of various customers, continuously improve service levels and quality, and adapt to changes in industrial structure at different stages. The demand for logistics services varies among different industrial structures, such as productive real economies and service-oriented economies. Taking the productive real economy as an example, it mainly includes manufacturing, construction, mining, etc. Its logistics service demand is mainly reflected in the transportation, warehousing, distribution, and other aspects of raw materials and finished products. The requirements for logistics services are mainly focused on improving transportation efficiency and reducing transportation costs. In industries dominated by service-oriented economy (such as commerce, finance, information, etc.), the demand for logistics services is more reflected in providing supply chain services close to consumers, logistics informatization, circulation and processing, etc. In order to meet the needs of different industries, logistics enterprises need to increase their efforts in technological innovation and service upgrading, adapt to the requirements of different industrial structures, and promote the high-quality development of the logistics industry.

However, in existing research, there is relatively little research on the adaptability and interrelationships between the logistics industry and industrial structure, and the research on the development strategies of logistics enterprises under different industrial structures is not in-depth enough. The logistics industry faces different development challenges and opportunities under different industrial structures, and further research is needed on how to formulate targeted development strategies based on different industrial structures. It is also the main research content of this article.

This paper started from the measurement of the high-quality development level of the logistics industry and the economy, discussed the impact of the high-quality development of the logistics industry on the high-quality economic development, and analyzed it under different threshold levels of the industrial structure. By exploring the relationship between high-quality development of the logistics industry and high-quality economic development, and empirically analyzing their differences in different stages of industrial structure development, it aims to provide strategic suggestions for the development of the logistics industry in different stages of high-quality economic development. The rest of this paper is arranged as follows. The Section 2 is the relevant literature and hypotheses, the Section 3 is the methodology, the Section 4 is the results, and the Section 5 is the conclusion and discussion.

## Literature review and hypotheses development

The logistics industry is a comprehensive service industry that includes a series of activities such as transportation, warehousing, freight transportation, information and others. It involves the movement of products from the origin to the production system, and then to their final consumption point to meet the needs of customers or enterprises [8]. It is a basic, strategic and leading industry that supports the development of the national economy. In recent years, with the advancement of technology, economic globalization, and rapid development of the logistics industry, domestic and foreign scholars have conducted extensive research on the relationship between the logistics industry and economic development. The promotion of high-quality development of the logistics industry on high-quality economic development has been widely recognized, and the industrial structure has also been one of the research objects. However, most studies focus on the impact of industrial structure on economic development levels, exploring how to improve economic quality through industrial structure upgrading, and how to optimize and adjust industrial structure to promote healthy market development. Research related to the logistics industry mainly focuses on the impact of industrial structure on high-quality development, the relationship between regional industrial structure and logistics resource advantages, and how to achieve the transformation of the logistics industry by adapting to industrial structure adjustments. However, studies focusing on the differences in the impact of high-quality development of the logistics industry on high-quality economic development at different levels of industrial structure are still lacking. This paper aims to address this gap and conduct research on this issue.

As an emerging complex industry, modern logistics has developed rapidly in recent years and plays an important role in guiding production and promoting consumption. In the short term, the concentration of the logistics industry can optimize the industrial structure through professional advantages and improve the core competitiveness of enterprises, the development of the logistics industry can promote the rationalization of the industrial structure in the regional sustainable development [9]. In the long run, the logistics industry is regarded as an effective tool for employment and economic sustainability, and logistics development may be an important factor in promoting long-term economic growth by injecting policies [10]. However, for the logistics industry, transportation and warehousing are obviously core activities, which will lead to a large consumption of primary and secondary energy, such as coal, gasoline, fuel, electricity, and natural gas. Therefore, it is also an important source of energy conservation and emission reduction [11]. It can be said that the rapid development of the logistics industry has greatly promoted the economic development. On the other hand, with the expansion of the logistics scale, it has brought a series of problems such as energy consumption, environmental pollution, urban traffic congestion, etc. The conflict between the traditional logistics development model and high-quality development is becoming more and more obvious. So, the important relationship between energy conservation and emission reduction in the logistics industry and high-quality economic development has also received extensive attention [7]. The economic development and logistics growth have accelerated the emission of carbon dioxide [12, 13]. Under the background of energy-efficient and emission reduction, reducing carbon emissions of logistics, promoting low-carbon development of logistics, and improving the efficiency of logistics operation are the focus of China’s energy conservation and emission reduction development, and also an effective way to accelerate the transformation of economic development model [13].

In view of these, the following hypothesis was proposed:

**H1**. The high-quality development of the logistics industry has a positive effect on the high-quality economic development.

Due to China’s economic policy and market demand, the focus in recent years has been to optimize the industrial and economic structure, adjust the economic development model, and strengthen regional connectivity. Advanced industrial structure, resource endowment and environmental regulation are conducive to reducing environmental pollution [1, 14]. China’s current economic development mainly depends on the manufacturing industry, and the economic activities of the manufacturing industry are an important source of carbon dioxide emissions [15]. It is estimated that in recent years, the carbon dioxide emissions of the manufacturing industry have reached 40% [16]. The high-quality development of the manufacturing industry is the main driving force of high-quality economic development and plays an important role in ensuring stable economic growth. And industrial structure adjustment is the driving force for the evolution of energy consumption structure [17], industrial structure optimization is also an effective way to reduce carbon dioxide emissions [18]. At the same time, the transformation and upgrading of manufacturing industry mainly includes the upgrading of value chain and industrial structure [19]. The upgrading of the industrial chain structure of the manufacturing industry is conducive to promoting the domestic economic cycle, smoothing the internal links of the industry, realizing the positive interaction between supply and demand, and improving the stability and competitiveness of the industrial chain. At the same time, it is of great practical significance to promote the transformation and upgrading of the industrial structure of the manufacturing industry and achieve a win-win situation of carbon emission reduction and economic development [19]. Therefore, under the goal of reducing carbon emissions, optimizing the industrial structure is an important way to rationally allocate and accelerate the resource cycle, and to achieve high-quality economic development [19].

The industrial structure refers to the allocation of economic resources in a country or region, which largely determines the utilization efficiency of such resources. The evolution of a region’s industrial structure is closely related to its economic growth, with the rationalization and upgrading of suchl structure having a positive effect on macroeconomic indicators [20]. The process of national or regional economic growth is a dynamic and evolutionary one characterized by the transformation of the industrial structure from an uncoordinated to a coordinated state and from a low-level to a high-level coordination, that is, it is a process of continuous optimization of the industrial structure [21]. While China’s industrial structure has been gradually developing, issues remain between different industrial structures: firstly, the proportion of the three industrial structures is uncoordinated; and secondly, the regional development of the three main industrial structures is unbalanced [22]. As of 2022, the proportion of the three industries in China’s GDP was 7.3:39.9:52.8. In comparison, the United States had a ratio of 1.13:18.55:80.31, while Germany’s ratio was 1.25:29.39:69.36. The experience of developed countries or regions shows that an advanced industrial structure is an effective means of promoting economic transformation and high-quality growth [23]. Nonetheless, the impact of rationalization and upgrading of an industrial structure on economic growth varies [20, 24]. At the same time, when the degree of industrial structure rationalization differs, the impact of an industrial structure upgrade on economic growth differs significantly [23, 25].

The optimization of the industrial structure can significantly promote the development of the logistics industry, and the upgrading of the industrial structure can effectively increase logistics demand, and is a realistic path towards achieving high-quality development in the logistics industry. On the one hand, rationalizing the industrial structure can strengthen the cooperation between upstream and downstream enterprises in the supply chain through the inter-industry linkage effect, and promote the growth of supply and demand in the logistics industry [26]. At the same time, rationalizing the industrial structure can also help to reduce energy consumption in logistics operations and play a role in low-carbon emission reduction, thereby improving the overall low-carbon efficiency of the logistics industry [27]. On the other hand, upgrading the industrial structure involves adjusting the position of the three sectors in the national economy, and gradually shifting the proportion of industrial structure advantages to capital-intensive, knowledge-intensive, and technology-intensive industries. Among the three industrial structures, the tertiary industry plays a significant role in promoting high-quality development in the logistics industry [28]. Furthermore, from a long-term perspective, continuously improving logistics infrastructure construction and providing high-quality logistics services can promote the adjustment of industrial structure towards the tertiary industry [29]. The logistics industry plays a crucial role in industrial agglomeration, enabling the integration of decentralized resources. This is especially true in the service industry, which can significantly promote the upgrading of the industrial structure [30].

In view of these, the following hypothesis was proposed:

**H2**. The impact of high-quality development level of logistics industry on high-quality economic development is different with different levels of industrial structure development.

In addition, environmental regulation can promote the innovation of environmental protection technology and energy-saving technology, promote green and clean energy to replace fossil energy, promote renewable energy to replace fossil energy, and promote low energy consumption and low emission industries to be replaced by high energy consumption and low emission industries, thus reducing their carbon emissions and promoting high-quality economic development [31]. Openness can also reduce carbon emissions, expand market boundaries, and help industries optimize resource allocation in a wider market [11]. Moderate government intervention is conducive to promoting the operation of the economic market, and excessive government intervention, as well as government corruption and market segmentation, can have negative impacts [32]. Urbanization can solve environmental problems by concentrating population, stimulating innovation and increasing wealth to promote sustainable economic development [33]. The demographic dividend is particularly important for the high-quality economic development of a region. With the continuous deepening of industrialization and urbanization in China, air pollution has become the most serious environmental problem, threatening the health and sustainable development of residents [1, 34]. The rapid growth of China’s economy and industrial upgrading have led to major changes in the employment structure, which often requires more skilled labor [35]. Moreover, technological innovation has a significant impact on the industry’s carbon emission reduction and high-quality economic development, and its marginal impact is on the rise [11].

In conclusion, while many studies have confirmed the close relationship between the logistics industry and economic development and have also discussed the impact of industrial structure on the logistics industry and high-quality economic development, the effect of the logistics industry on high-quality economic development at different stages of industrial structure development, rationalization, and upgrading still requires further investigation. Therefore, this paper aims to how the high-quality development of the logistics industry affects high-quality economic development during different stages of industrial structure development and to develop appropriate strategies accordingly. This research employs the benchmark regression model to analyze the impact of the logistics industry’s high-quality development on high-quality economic development, and the panel threshold model to analyze the impact of the logistics industry on high-quality economic development at different levels of industrial structure development.

## Methodology

### Model design

#### Benchmark regression model

In order to investigate the impact of high-quality development of the logistics industry on high-quality economic development, it constructed a panel model to investigate. The specific estimation model is as follows:

$${\text{lneq}}_{i,t}=\alpha_{1}+\beta_{1}{\text{lnlq}}_{i,t}+\gamma_{1}{\text{X}}_{i,t}+\mu_{i}+\varepsilon_{i,t}$$ (1)

Where, the dependent variable lneq represents the high-quality development level of the regional economy, the core independent variable lq represents the high-quality development level of the logistics industry in various regions, and X is the control variable, including the environmental regulation level er, the openness level op, the government intervention gi, the urbanization level ur, and the human capital hu. i represents region i, t represents year, μ_*i*_ represents unobserved individual effects, ε_*i*,*t*_ is a random disturbance term. In addition, in this model, in order to avoid violent fluctuations in data and eliminate possible heteroscedasticity, the relevant time series data were logarithmized.

#### Panel threshold model

To investigate whether the high-quality development of the logistics industry has nonlinear effects and threshold conditions on high-quality economic development, the threshold regression model proposed by Hansen was used to construct the threshold effect model for high-quality development of the logistics industry and its impact on high-quality economic development. If there is only one threshold, use the single threshold model (2):

$${\text{lneq}}_{i,t}=\alpha_{2}+\beta_{2}{\text{R}}_{i,t}I(Q_{i,t}\leq q)+\delta_{2}{\text{R}}_{i,t}I(Q_{i,t}>q)+\gamma_{2}{\text{X}}_{i,t}+\mu_{i}+\varepsilon_{i,t}$$ (2)

Where, R represents the threshold independent variable and the core independent variable, which refers to the level of high-quality development of the logistics industry; I(·) is the indicative function; while Q represents the threshold variable, which includes both the level of industrial structure upgrading and the degree of industrial structure rationalization; q represents the threshold value;

β_2_ is the impact coefficient of the high-quality development of logistics industry on high-quality economic development when *Q*_*i*,*t*_ ≤ *q*; *δ*_2_ is the impact coefficient of the high-quality development of logistics industry on high-quality economic development when *Q*_*i*,*t*_ > *q*; when *β*_2_ ≠ *δ*_2_, there is a threshold effect, otherwise there is no threshold effect. If there are two thresholds, the model formula (2) can be expanded to the double threshold model formula (3):

$${\text{lneq}}_{i,t}=\alpha_{3}+\beta_{3}{\text{R}}_{i,t}I(Q_{i,t}\leq {\text{q}}_{1})+\delta_{3}{\text{R}}_{i,t}I({\text{q}}_{1}<Q_{i,t}\leq {\text{q}}_{2})+\rho_{3}{\text{R}}_{i,t}I(Q_{i,t}>{\text{q}}_{2})+\gamma_{3}{\text{X}}_{i,t}+\mu_{i}+\varepsilon_{i,t}$$ (3)

Where, q_1_ and q_2_ are two thresholds, and q_1_<q_2_, these two thresholds divide the entire sample into three intervals; β_3_, *δ*_3_ and *ρ*_3_ represent the influence coefficients of the core explanatory variable R on the dependent variable lneq in the three different intervals. If there are 3 or more thresholds, this method can still be used to establish a multi threshold model.

### Variable selection

#### Dependent variable

The dependent variable of this paper is the level of high-quality economic development (eq). The connotation of this indicator is based on the research recognized by many scholars, with reference to previous research by Yan and others [36–38], it is measured by using the five indicators of innovation, coordination, green, openness and sharing, as well as the indicator system established by the indicator and the corresponding secondary indicators. The indicator system follows the previous research [36–38].

#### Core independent variable

The core independent variable in this paper is the high-quality development level of the logistics industry. The indicator system, which is based on previous studies [36–38], employs the input-output indicator system to measure its value. On this basis, a Super-SBM (Slacks-based Measure) model was established that accounts for unexpected output, and the logistics industry high-quality development evaluation index system was then calculated using the MAXDEA ultra7.10 software.

#### Threshold variables

The threshold variables were expressed in terms of industrial structure, including the level of industrial structure upgrading and the level of industrial structure rationalization. The level of industrial structure upgrading was expressed in terms of the proportion of the output value of the tertiary industry and the output value of the secondary industry, the higher the value, the higher the level of industrial structure upgrading; The rationalization level of industrial structure was represented by the Thiel index, and the smaller the value is, the more reasonable the industrial structure is. In order to facilitate the analysis, the reverse standardization was made in the analysis [36–38].

#### Control variables

The level of environmental regulation (er), is expressed by the ratio of investment completed in industrial pollution control to industrial added value; Openness (op) is expressed by the ratio of Foreign Direct Investment to GDP; Government intervention (gi) is expressed by the proportion of local fiscal expenditure in GDP; Urbanization level (ur) is expressed by the proportion of the urban population in the total population; Human capital (hu) is expressed by per capita education expenditure [36–38].

### Data source and description statistics

Since the data of Tibet, Hong Kong, Macao and Taiwan are partially missing, this paper selected the data of the remaining 30 provinces and cities in China from 2001 to 2019 for research. The data were mainly from China Statistical Yearbook, China Energy Statistical Yearbook, China Environmental Statistical Yearbook. If some original data were missing, the interpolation method shall be used to make up (the same below). The description statistics of main variables are shown in Table 1.

**Table 1:** Statistical characteristics of main variables.

Category	Variable	Obs	Mean	Std.Dev.	Min	Max
Dependent variable	eq	570	0.420	0.091	0.198	0.715
Independent variable	lq	570	0.429	0.265	0.110	1.366
Threshold variables	isa	570	1.149	0.604	0.527	5.234
	isr	570	1.062	0.364	0.233	2.553
Control variables	er	570	59.064	53.501	1.610	470.890
	op	570	0.026	0.024	0.000	0.163
	gi	570	0.219	0.106	0.080	0.760
	ur	570	51.376	14.759	20.350	89.607
	hu	570	1685.855	1252.857	177.090	11421.980

## Results

### Benchmark regression results

Based on China’s provincial panel data from 2001 to 2019, it used the measurement software Stata for empirical analysis. The estimation method was selected according to the results of the F test and Hausman test in Stata, where the F statistic is 195.52 (the corresponding P-value is 0.0000), indicating that each province, autonomous region and city has different regional effects on high-quality economic development. Hausman statistic is 27.84 (corresponding P-value is 0.0001), which strongly rejects the original hypothesis that the regional impact effect is a random effect, indicating that the fixed effect model (FE) is more effective for estimation. The regression estimation results are shown in Table 2. Model (1)a regresses the core independent variable, and model (1) adds control variables for regression.

**Table 2:** Benchmark regression analysis results.

Variables	Model(1)a	Model(1)
lnlq	0.100***	0.019**
	(0.016)	(0.009)
lner		-0.010**
		(0.004)
lnop		0.021***
		(0.005)
lngi		-0.095***
		(0.026)
lnur		0.273***
		(0.046)
lnhu		0.080***
		(0.014)
_cons	-0.791***	-2.534***
	(0.018)	(0.130)
N	570	570
adj. R2	0.061	0.667

Standard errors in parentheses

* ** *** represents the significance levels of 10%,5% and 1% respectively.

From the regression results of model (1)a and model (1) in Table 2, it can be found that with the introduction of control variables, the coefficient of the core independent variable lnlq is always significantly positive at the level of 5%. From an economic perspective, the higher the level of high-quality development of the logistics industry, the greater its role in promoting the high-quality development of the region’s economy, which confirms the hypothesis 1 in this theoretical analysis. Firstly, the logistics industry plays an important role in production and circulation, and high-quality development of the logistics industry can enable more reasonable utilization of resources, thereby promoting high-quality economic development. Secondly, high-quality development of the logistics industry can improve the supply chain, reduce logistics costs and time, and improve the transparency and efficiency of the supply chain, which is beneficial for the production and sales of enterprises, as well as the transportation of goods, reducing waste and improving efficiency. Thirdly, the logistics process not only involves transportation and warehousing, but also includes information, funds, and other content, especially in the context of rapid development of e-commerce, the logistics industry has made an increasing contribution to market exploration and expansion, developing a competitive logistics industry can bring more business opportunities and strengthen the commercial foundation of the region. In addition, the high-quality development of the logistics industry will drive the development of related industries, especially the service industry. In China, the storage and transportation equipment and technology industry, cross-border e-commerce industry, engineering equipment and other industries are also inseparable from the logistics industry. Therefore, the high-quality development of the logistics industry plays an important role in promoting the high-quality development of the regional economy. Improving the various indicators of the logistics industry will have a positive impact on the high-quality development of the regional economy.

Based on the results of model (1), the regression results of control variables reveal that certain control variables have a significant positive impact on high-quality economic development. For instance, the level of openness has a significant positive impact on high-quality economic development. In addition, the coefficient of urbanization level is also significantly positive, indicating that promoting the urbanization process is an important means of improving the level of high-quality economic development. Moreover, the coefficient of human capital level is significantly positive, which corroborates that talent is an important driver of high-quality economic development. However, the coefficient of environmental regulation level is negative, suggesting that the more severe environmental problems are, the greater their impact on the level of high-quality economic development. Furthermore, the coefficient of government intervention level is also negative, indicating that a larger government and stronger government intervention in the market may lead to unhealthy market operations, thereby impeding the high-quality economic development.

### Threshold effect results

Consider the impact of changes in industrial structure on the relationship between the high-quality development of logistics industry and high-quality economic development when taking different values. The advanced level of industrial structure and the rationalization level of industrial structure are set as threshold variables, the threshold values of model (2) and model (3) are estimated, and a significance test based on the threshold values is conducted to determine the existence of the threshold effect. The statistical software stata16 is employed for repeated sampling for 300 times, and the single threshold, double threshold, and triple threshold tests are subsequently carried out. The results are shown in Table 3.

**Table 3:** Threshold inspection results.

Threshold variable	Threshold number	F	P-value	Critical Value			Threshold	95% CI	
				10%	5%	1%			
lnisa	Single	86.840	0.000	34.849	38.967	51.673	-0.002	-0.003	0.001
	Double	29.210	0.063	23.667	32.181	42.235	0.281	0.269	0.281
	Triple	7.410	0.753	22.269	27.139	33.884	-	-	-
lnisr	Single	85.680	0.000	35.217	42.088	56.370	0.087	0.067	0.105
	Double	53.890	0.000	30.183	35.727	47.475	0.229	0.222	0.230
	Triple	22.450	0.867	63.137	73.476	93.136	-	-	-

In model (2), the P-value of the F statistic used for the single threshold significance test is 0.000, which is significantly less than 0.05, indicating that model (2) has a significant single threshold effect, necessitating further testing of the double threshold effect. Since the P-value of the F statistic for the double threshold significance test is 0.063, which is dignificantly less than 0.1, it indicates that the model (2) demonstrates a significant double threshold effect, so it is necessary to further test the triple threshold effect. The P-value of the F statistic for the triple threshold significance test is 0.753, which is far greater than 0.1, indicating that there is no triple threshold effect in model (2). As such, the advanced level of industrial structure has obviously passed the single threshold and double threshold but not the triple threshold, so the double threshold is selected.

In model (3), the P-value of F statistic used for the single threshold significance test is 0.000, significantly less than 0.05, indicating that model (3) has significant single threshold effect, so further double threshold effect test is required. Since the P-value of the F statistic of the double threshold significance test is 0.000, which is significantly less than 0.05, indicating that the model (3) has a significant double threshold effect, so it is necessary to further test the triple threshold effect. Since the P-value of the F statistic of the triple threshold significance test is 0.867, which is far greater than 0.1, indicating that there is no three threshold effect in model (3). That is, the degree of industrial structure rationalization has significantly passed the single and double thresholds but not the triple threshold, so the double threshold is selected.

The significant presence of the threshold effect demonstrates that the improvement of the quality development level of the logistics industry has different effects on the quality development of the economy at different stages of the industrial structure’s development.

The regression results of threshold effect are shown in Tables 4 and 5.

**Table 4:** Regression results of threshold variable lnisa.

lneq	Coef.	Robust Std. Err.	t	P>t	[95% Conf. Interval]	
lner	-0.005	0.005	-1.020	0.314	-0.016	0.005
lnop	0.023	0.009	2.420	0.022	0.004	0.042
lngi	-0.090	0.045	-1.990	0.056	-0.182	0.002
lnur	0.227	0.067	3.370	0.002	0.089	0.365
lnhu	0.077	0.022	3.570	0.001	0.033	0.121
lnisa≤-0.002	0.047	0.020	2.390	0.024	0.007	0.087
-0.002<lnisa≤0.281	0.004	0.017	0.230	0.816	-0.030	0.038
lnisa>0.281	-0.030	0.018	-1.680	0.104	-0.067	0.007
_cons	-2.344	0.283	8.280	0.000	-2.923	-1.765

**Table 5:** Regression results of threshold variable lnisr.

lneq	Coef.	Robust Std. Err.	t	P>t	[95% Conf. Interval]	
lner	-0.007	0.006	1.200	0.241	-0.018	0.005
lnop	0.026	0.008	3.110	0.004	0.009	0.043
lngi	-0.078	0.041	1.920	0.065	-0.162	0.005
lnur	0.251	0.076	3.290	0.003	0.095	0.407
lnhu	0.072	0.025	2.880	0.007	0.021	0.123
lnisr≤0.087	0.090	0.025	3.540	0.001	0.038	0.142
0.087<lnisr ≤0.229	0.048	0.019	2.490	0.019	0.009	0.088
lnisr>0.229	0.005	0.018	0.270	0.789	-0.032	0.042
_cons	-2.345	0.247	9.480	0.000	-2.851	-1.840

For the threshold variable, which is the advanced level of industrial structure, when lnisa ≤ -0.002, the influence coefficient of lnisa on lneq is 0.047, and it passes the 5% significance test; When -0.002<lnisa≤0.281, the influence of lnisa on lneq is not significant; When lnisa>0.281, the influence coefficient of lnisa on lneq is negative, but not significant at the 10% level, significant at the 15% level. This shows that at a low level of industrial structure, the high-quality development of the logistics industry promotes high-quality development. Firstly, the logistics industry’s high-quality and efficient services improve logistics efficiency by delivering production factors and consumer goods quickly to various locations, reducing delivery times, operating costs, and enhancing the competitiveness of enterprises. Secondly, the high-quality development of the logistics industry strengthens social connections and exchanges, bridging regional economic disparities, increasing employment opportunities, and improving people’s living standards. Notably, high-quality logistics development promotes the implementation of green logistics strategies, contributing to reducing pollution and environmental damage in logistics areas, and achieving sustainable development goals. Furthermore, the logistics industry provides infrastructure support for the construction of modern markets and supply chains, cultivating modern management concepts, promoting economic development upgrades, and building market entities.

When the level of the industrial structure is further improved, it will reduce the dependence of high-quality economic development on industries and other entities, thus reducing the dependence on the logistics industry. With the high-quality economic development, the internal coordination and optimization management of various major industries will be further optimized and improved. In this situation, although the logistics industry is still an important link that runs through the entire production process, its role in promoting high-quality economic development may be relatively small. However, the logistics industry still needs to maintain high-quality development and maintain its own competitiveness. The level and quality of logistics industry services can be continuously improved through industrial chain integration, service quality improvement, technological innovation, and other means. In addition, the logistics industry can actively expand into new fields, such as new energy and cross-border e-commerce, in order to open up new market space. By continuously providing high-quality logistics services, the logistics industry can still contribute to the high-quality economic development.

When the threshold variable is the level of industrial structure rationalization, when lnisr≤0.0087, the influence coefficient of lnisr on lneq is 0.090, passing the test at the 1% significance level; When 0.087<lnisr ≤ 0.229, the influence coefficient of lnisr on lneq is 0.048, passing the test at the 5% significance level; When lnisr>0.229, the effect of lnisa on lneq is not significant. These results demonstrate that when the rationalization of the industrial structure is low, the high-quality development of the logistics industry plays a role in promoting high-quality economic development. However, as the degree of rationalization of the industrial structure increases, it reduces the dependence of high-quality economic development on industries and other entities, resulting in lower dependency on the logistics industry. When the rationalization of the industrial structure is relatively low, the logistics industry promotes the flow of production factors, optimizes resource allocation, and boosts high-quality economic development through refined management of the logistics process chain by improving logistics efficiency and optimizing service systems. As the degree of rationalization of the industrial structure increases, the economy becomes more mature, and the impact of the high-quality development of the logistics industry on the high-quality economic development decreases. Firstly, when the industrial structure is at a lower level, the high-quality development of the logistics industry has greater potential for cost reduction. As the degree of rationalization increases, the impact of the logistics industry on cost reduction reduces, and emphasis shifts towards improving efficiency and service quality. Secondly, as the degree of rationalization of the industrial structure increases, the service supply and logistics service quality in the market typically increase, and the market form changes that can be achieved through improving logistics services alone are limited. Moreover, as the degree of rationalization of the industrial structure increases, the interdependence between different industries strengthens, collaboration and cooperation between various links in the supply chain and industrial chain improve, and optimization of the logistics circulation link efficiency steadily advances, indicating the gradual reduction of the impact of the logistics industry on the industrial structure. Additionally, from the perspective of the domestic demand of the economy, as high-quality economic development and rationalization of the industrial structure progress, the market demand structure may also change. This may lead to a decrease in the scale and demand of existing logistics businesses. To promote high-quality economic development, the logistics industry needs to seek new growth points through innovative services and expanding service areas such as international logistics, as well as logistics services in new industries and formats.

### Robust test

In order to further verify the reliability of the relationship model between the high-quality development of the logistics industry and the high-quality economic development, it used the added value of the logistics industry as the independent variable and conducts regression analysis on the proxy variable of high-quality development of the logistics industry, the results are shown in Table 6. According to the regression results, after using the added value of the logistics industry as the proxy variable, the relationship between the high-quality development of the logistics industry and the high-quality economic development has not changed substantially. Its significant role in promoting the development is still rational, and the coefficient size is almost the same as the previous results. Therefore, it can be judged that the benchmark regression results in this paper are robust.

**Table 6:** The robustness of regression results.

	(1)		(2)
	Model(4)		Model(1)
lnwlzjz	0.033**	lnlq	0.019**
	(0.016)		(0.009)
lner	-0.009**	lner	-0.010**
	(0.004)		(0.004)
lnop	0.021***	lnop	0.021***
	(0.005)		(0.005)
lngi	-0.101***	lngi	-0.095***
	(0.026)		(0.026)
lnur	0.268***	lnur	0.273***
	(0.046)		(0.046)
lnhu	0.060***	lnhu	0.080***
	(0.018)		(0.014)
_cons	-2.615***	_cons	-2.534***
	(0.135)		(0.130)
N	570	N	570
adj. R^2^	0.667	adj. R^2^	0.667

Standard errors in parentheses

* ** *** represents the significance levels of 10%,5% and 1% respectively.

## Conclusion and discussion

This paper first analyzed the positive impact of high-quality development of the logistics industry on high-quality economic development. The higher the level of high-quality development of the logistics industry, the greater its role in promoting high-quality economic development in the region. At the same time, it is also found that the level of openness has a significant positive impact on high-quality economic development, indicating that foreign trade cooperation can help improve the level of high-quality economic development. The coefficient of urbanization level is also significantly positive, indicating that promoting the urbanization process is an important way to improve the level of high-quality economic development. The coefficient of human capital level is significantly positive, which confirms that talent is an important driving force to drive high-quality economic development, and improving the quality of labor force and increasing investment in education are conducive to improving high-quality economic development. The coefficient of the level of environmental regulation is negative, which means that the more environmental investment, the more serious the environmental problem is, which increases the national expenditure and is not conducive to high-quality economic development. The coefficient of government intervention level is negative, which indicates that the government is the maintainer of the market environment, not the participant. Therefore, excessive government expenditure will squeeze out the utility of enterprises, universities and other market players. Excessive intervention in the market will lead to unhealthy operation of the market, thus hindering high-quality economic development. The above conclusions are basically consistent with existing research. Secondly, this paper also verified that the impact of the high-quality development level of logistics industry on the high-quality development of economy varies with the development level of industrial structure. When the industrial structure is at a low level, that is, when it mainly depends on the secondary industry, the high-quality development of the logistics industry promotes the high-quality development; With the gradual optimization of the industrial structure, the impact of high-quality development of the logistics industry on high-quality economic development is no longer significant. This conclusion will provide new ideas for subsequent research on the high-quality development of the logistics industry and other industries.

Based on the research conclusions, the following suggestions were proposed:

First, adhere to openness, increase investment in education, and adhere to scientific and technological innovation as the primary productive force. We will continue to realize further economic development through opening to the outside world. Human capital is the most important part of innovation activities. All regions should attach great importance to the allocation of human resources, promote the inflow of scientific and technological talents, stimulate innovation vitality, and use the agglomeration effect of high-quality human capital to provide talent guarantee for high-quality economic development.

Second, optimize the industrial structure. Overdependence on the secondary industry is not conducive to high-quality economic development in the long run. It is necessary to give full play to the structural dividend, promote the rational allocation of industrial sectors, limit the development of high pollution, high energy consumption and high emission industries, promote the development of green, low-carbon, recycling and high-tech industries, and promote the green and low-carbon transformation of economic development and reduce carbon emissions through industrial and regional links. Promote the play of industrial structure correlation effect and structural effect, and improve the rationalization level of industrial structure; Promote the upgrading effect and innovation effect of industrial structure, and improve the advanced level of industrial structure. Within the industry, it is necessary to enhance the learning of new technologies, constantly improve the quality of products, so as to enhance the competitiveness of the industry, ensure the continuous improvement of the industrial structure, and achieve high-quality economic development.

Third, clarify the government’s positioning, highlight market advantages and pay attention to regional differences. In the process of high-quality economic development, the government is both a leader and a manager. It should clarify its own position, appropriately delegate power to the market, encourage market-oriented reform, provide a good economic environment, establish sound rules and regulations, scientifically and systematically supervise the market, and ensure high-quality economic development. Each province should break administrative barriers and geographical constraints, build a scientific, dynamic and fair high-quality development performance evaluation system based on the differences in geographical characteristics, resource endowment, economic development and logistics development level of each province’s location, reasonably balance regional interests, and gradually reduce regional differences.

Fourth, further promote the deep integration and development of logistics and related industries. Deeply study and analyze the laws of industrial development research, and promote the collaborative agglomeration and integrated development of logistics industry, manufacturing industry, commercial circulation industry and other industries to improve the operational efficiency of logistics industry. By extending warehousing, transportation and other functional logistics services, to promote the transformation of logistics business to comprehensive supply chain services, further enhance the breadth and depth of the integration of logistics industry into the industrial chain and supply chain, significantly improve the efficiency of supply chain organization, and realize the coordinated development of industry while building an independent, safe and controllable supply chain system. And each region should focus on promoting industrial integration and development to improve the high-quality development level of regional economy. For example, in areas dominated by traditional industries, the demand of industry for logistics industry and the supporting role of logistics industry for industry have not been fully played. It is necessary to further improve the development level of each industry, and develop the matching logistics industry around the development priorities of each industry. In areas with relatively developed related industries, the logistics industry supporting them has also reached a high level, the next step should focus on improving the level of integrated development of logistics and related industries.

Fifthly, it is essential to continue promoting the high-quality development of the logistics industry. To achieve the positive impact of high-quality logistics on high-quality economic development, different development strategies must be developed for different levels of the industrial structure. When the level of industrial structure is low, the logistics industry must prioritize developing and improving the quality of its services to promote high-quality economic development. This can be achieved by prioritizing the following aspects: (1) Improving logistics efficiency and service quality: The logistics industry must optimize logistics networks, improve logistics management, and promote logistics information technology to provide efficient, low-cost, and high-quality logistics services. (2) Reducing logistics costs: Logistics costs directly affect the production and development of the entire industry, and measures to reduce logistics costs must be prioritized. This can be achieved by improving supply chain management, optimizing logistics processes, promoting the application of technologies such as the Internet of Things and artificial intelligence, and strengthening logistics enterprise management and standardized construction. (3) Building a logistics platform: The logistics platform is an important carrier for connecting different industries and optimizing the allocation of logistics resources, and can achieve the goals of logistics information sharing and logistics resource reuse. Logistics platforms are generally jointly built by the government, logistics enterprises, and relevant industrial organizations, and can also be operated digitally and online through technological means such as the Internet and the Internet of Things. When the industrial structure is at a high level, the high-quality development of the logistics industry continues to play a crucial role in the high-quality development of the economy. Effective methods to achieve this include: (1) Utilizing information technology to promote digital construction: Through the application of information technology, achieve visualization and intelligence of logistics networks and data, optimize and strengthen existing logistics supply chains, and make information sharing between all links of the logistics industry closer. (2) Optimizing resource allocation and coordination: By strengthening coordination and cooperation among various links in the supply chain, the synergy effect of logistics distribution, marketing, and other functions can be achieved. Continuously optimizing resource allocation, improving logistics efficiency, and improving consumer satisfaction can drive high-quality development of the industrial chain. (3) Improving logistics facilities and service models: In a high-level industrial structure, emerging service models become more important. Logistics enterprises must plan logistics facilities and storage resources rationally, utilize technologies such as cloud computing and big data, and improve market share and revenue by providing high-quality services and user experiences tailored to consumers. (4) Promoting green logistics: Green logistics has become a visionary and important trend. In addition to energy conservation and emission reduction, green logistics can bring more convenience for coordination in transportation networks, strengthen environmental protection, avoid pollution and health impacts, and improve the image of logistics enterprises and customer satisfaction. In summary, the high-quality development of the logistics industry requires the formulation of corresponding strategies and plans based on the characteristics of the industrial structure to promote high-quality economic development. The logistics industry must adapt to changes in market demand and technological developments, continuously strengthen technological innovation and refined management, and provide more high-quality services to adapt to the development and market challenges of the new era.

This article conducts empirical analysis on the relationship between the logistics industry and high-quality economic development at different levels of industrial structure. However, it has not explored the direction of high-quality development in the future logistics industry, and what kind of logistics industry can continue to promote high-quality economic development in the future. This will be the focus of the next research step.
